# Impact of a Palladium(II)-tris(2-carboxyethyl)phosphine Complex on Normal Cells: Toxicity and Membrane Interaction

**DOI:** 10.3390/molecules30030476

**Published:** 2025-01-22

**Authors:** Hanna Pruchnik, Katarzyna Solarska-Ściuk, Anita Dudek, Aleksandra Włoch

**Affiliations:** 1 Department of Physics and Biophysics, Wrocław University of Environmental and Life Sciences, Norwida 25, 50-375 Wrocław, Poland; anita.dudek@upwr.edu.pl (A.D.); aleksandra.wloch@upwr.edu.pl (A.W.); 2 Faculty of Biotechnology, Collegium Medicum, University of Rzeszow, Pigonia 1, 35-310 Rzeszow, Poland; ksolarska@ur.edu.pl

**Keywords:** palladium(II) complex, tris(2-carboxyethyl)phosphine, cytotoxicity, HMEC-1, erythrocytes, membrane, biophysical study

## Abstract

Palladium(II) complexes with tris(2-carboxyethyl)phosphine (PdTCEP) show promise for biomedical applications due to their distinct chemical characteristics. This study explored the toxicity of PdTCEP towards normal human cells and examined its interactions with model cell membranes. Two cell types were used to evaluate cytotoxicity: human microvascular endothelial cells (HMEC-1) and red blood cells (RBCs). In HMEC-1 cells, PdTCEP reduced survival to about 80% at 15 µM, with the most significant drop—down to 40%—occurring at 40 µM. The production of reactive oxygen species (ROS) increased in a manner dependent on both dose and time, especially after 72 h of incubation. Despite these effects, PdTCEP caused only minor hemolysis in RBCs, with hemolysis levels staying below 10% even at higher concentrations. Fluorescence anisotropy measurements showed that PdTCEP minimally affects the hydrophobic core of the lipid bilayer, with slight changes observed at concentrations above 40 µM. Generalized polarization (GP) analysis indicated a slight decrease in lipid polar head packing with increasing PdTCEP concentration. Complementary FTIR analysis supported these findings by providing detailed insights into PdTCEP-membrane interactions. This research underscores PdTCEP’s selective cytotoxicity and structural effects on membranes, suggesting its promise for more in-depth biological and pharmacological studies.

## 1. Introduction

Cancer poses a growing challenge around the globe. The World Health Organization reports show that cancer is the first or second leading cause of premature death (among people aged 30–69) in 134 countries out of 183 that participated in the analysis. Of the 15.2 million premature deaths recorded in 2016, 4.5 million, i.e., 29.8%, were caused by cancer. According to the latest estimates of the WHO, by 2050, the global burden of cancer will increase to 35 million [1,2]. Along with surgery and radiotherapy, chemotherapy is a mainstay of cancer treatment. Chemotherapy is the most frequently used systemic treatment for suppressing cancer cell proliferation, disease progression, and metastasis. Platinum-based drugs, namely cisplatin, carboplatin, and oxaliplatin are among the most effective anticancer drugs, which have been widely used. However, some drawbacks such as neurotoxicity, nephrotoxicity, intrinsic resistance of some tumors, and dose-limiting side effects limited the use of the platinum diamine compounds, cisplatin, and carboplatin [3,4,5,6,7].

To overcome these obstacles and develop safer and more effective countermeasures, intensive efforts are being made to design and pharmacologically evaluate other metal-based drugs. It has turned out that transition metal complexes (e.g., Ru, Rh, Ir) are also promising in terms of spectrum of action, reduced side effects, and overcoming resistance. Transition metals are promising as chemotherapy agents, some showing potencies and selectivity exceeding that of cisplatin. Interestingly, these new transition metal complexes exhibit a variety of mechanisms of action. Some bind to DNA, causing deformations and eventually apoptosis (analogous to cisplatin), other metallodrugs disrupt other vital processes in cells in order to kill target cancer cells, including binding to mitochondria, or disruption of the NAD+/NADH balance inside cells. Exploring these new mechanisms of action against cancerous cells has become a focus for the synthesis of the next generation of anticancer compounds [5,8,9], e.g., half-sandwiched iridium(III) compounds with Schiff bases obtained from *N*-phenylcarbazole or triphenylamine and 2-formylpyridine and 2-formylchinoline. These compounds were observed to bind to proteins and transport through serum protein, catalyze the oxidation of the nicotinamide adenine dinucleotide coenzyme, and increase reactive oxygen species levels in cells, which resulted in an antitumor mechanism of oxidation. Also, these compounds possessed an energy-dependent cellular uptake mechanism which allowed them to effectively accumulate in lysosomes, damage the integrity of acidic lysosomes, disrupt the cell cycle, induce a change in mitochondrial membrane potential, and eventually lead to apoptosis [6,10].

Another example of transition metal compounds exhibiting very interesting properties are platinum(II), rhodium(III), and iridium(III) complexes with tris(carboxyethyl)phosphine P(CH_2_CH_2_COOH)_3_. These compounds exhibit anticancer activity over several cancer lines, for example: Sk-mel (human, Caucasian, skin, melanoma), SH-4 (melanotic melanoma), Colo-829 (human, umbilical metastatis, melanoma), C-32 (amelanotic melanoma), MCF7 and T-47D (triple positive breast cancer), and MDA-MB-231 (triple negative breast cancer). The strongest activity they exhibit is against MDA-MB-231 cells. The efficacy of the Pd compound against MDA-MB-231 (IC_50_ = 8–10 µM) is greater than the efficacy of cisplatin (IC_50_ = 61 ± 7 µM) [11,12]. Moreover, the antitumor activity of the platinum(II) complex PtCl_2_(TCEP)_2_ was checked against canine (GL1, CL1, CLBL-1, D-17) and human (U2-OS) cancer lines. The results showed that PtTCEP is an active cytotoxic and antiproliferative agent against the above-mentioned cell lines, acting at concentrations several times lower than cisplatin [13]. The cytotoxicity of cisplatin originates from its binding to DNA and the formation of covalent cross-links. The 1,2-intrastrand d(GpG) cross-link is the major adduct. The binding of cisplatin to DNA causes significant distortion of the helical structure and results in the inhibition of DNA replication and transcription. The Pt^2+^ unit covalently binds to DNA, particularly to the N7 of either guanine (G) or adenine (A) in the nucleotide sequences GG and AG to form interstrand cross-links. The so-formed cisplatin-DNA unit activates a new cellular pathway which leads to transcription inhibition, cell-cycle arrest, DNA repair, and finally apoptosis [14,15,16]. It turned out that PtCl_2_(TCEP)_2_ induced an internal (mitochondrial) caspase-dependent path of apoptosis, which is typical also for other derivatives of platinum. Observed apoptosis stemmed from the lowering of Bcl-X_L_ protein’s expression and the increase in pro-apoptotic BAX protein. The compound caused activation of caspase 8, and to a lesser extent, activation of caspase 12 in D-17 and U2-O2 cells. PtCl_2_(TCEP) stopped cell cycle progression in the G2/M phase in canine osteosarcoma and human cancer cell lines (damage of DNA) [13]. Platinum complexes can react with a number of cell components, e.g., with glutathione or with blood plasma proteins like serum albumin (HSA). This can influence the efficiency of platinum compounds because a significant amount of Pt(II) complexes can be eliminated by binding to plasma proteins. In addition to this, cisplatin has a tendency to interact with phosphatidylserine and other phospholipid components of cell membranes, and, in this way, it modulates functions of the cell membrane [17,18]. The interaction of PtCl_2_(TCEP)_2_ with a simple lipid model and with plasmid DNA was investigated. The studies of PtCl_2_(TCEP)_2_/DPPC showed that, whereas the cisplatin tends to remain in the polar head group region causing a decrease in flexibility of the bilayer, the investigated new compound enters into the hydrophilic region of DPPC. The PtCl_2_(TCEP)_2_ causes condensation of the plasmid DNA [19]. Based on tests so far, another promising compound appears to be the palladium(II) complex, which has chemical similarities to platinum [20]. Our previous research shows that the palladium(II) complex with tris(2-carboxyethyl)phosphine-*trans*-[PdCl_2_(TCEP)_2_]-PdTCEP has anti-tumor activity against many cell lines, e.g., SK-mel (malignant melanoma), SH-4 (melanotic melanoma), Colo-829 (malignant melanoma), and C-32 (amelanotic melanoma). For some of them, e.g., MDA-MB-231 (triple negative breast cancer), the activity of PdTCEP (IC_50_ = 8.1 ± 6.7) is much higher than the activity of cis-platinum (IC_50_ = 63 ± 6.8). The above data indicate that PdTCEP has the potential for anticancer therapy, but to ensure the safety and efficacy of this compound, its toxicity and interactions with normal cells should be tested. Among other things, it should be investigated whether the palladium complex affects the integrity of cell membranes, which may lead to their destabilization and cell death. It is also important to investigate the effect of this compound on morphological components of blood, such as erythrocytes. To our knowledge, no studies have been conducted on the interactions between PdTCEP and red blood cells (RBCs) or their direct effects on biological membranes (RBCMs) and liposome models of membranes. Hence, the aim of the tests was to determine the hemolytic activity of the compound in relation to red blood cells and to investigate the effect on selected physicochemical parameters of erythrocyte and liposome membranes [21,22,23,24]. It was also important to check the effect of the tested complex on the integrity of the erythrocyte membrane by analyzing the lysis process of erythrocytes. Furthermore, the effect of PdTCEP on HMEC-1 cells was evaluated. HMEC-1 cells are often used as a cellular model in in vitro studies on vascular biology and in testing new drugs. They facilitate the examination of interactions between endothelial cells and other cell types and the analysis of the effect of various substances on the function of blood vessels [25]. In our studies, the HMEC-1 cell line was used as a model system to investigate the toxicity of PdTCEP in relation to human dermal microvascular endothelial cells. The cytotoxicity findings obtained from the MTT assay were corroborated using a fluorimetric method involving the Hoechst 33258 fluorescent probe. This technique enabled the quantification of cell viability by measuring DNA content, providing an additional layer of validation for the observed results.

## 2. Results

### 2.1. Immortalized Human Microvascular Endothelial Cells

#### 2.1.1. MTT Test and DNA Content

**Figure 1:**
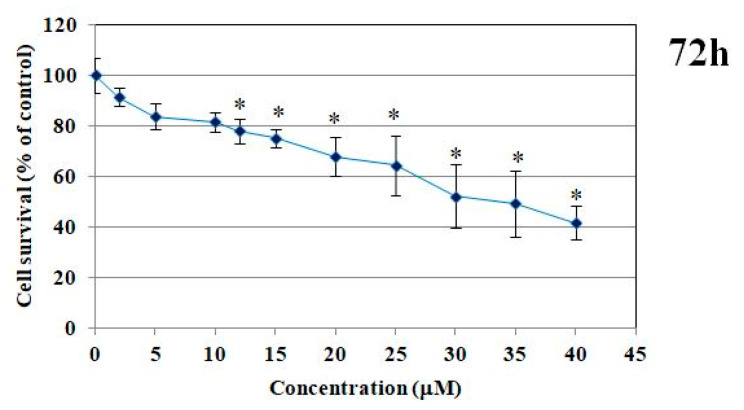
Cytotoxicity of HMEC-1 induced by PdTCEP. Cell survival of HMEC-1 cell line of PdTCEP was measured by MTT assay after 72 h exposure. Statistical evaluation of differences was made using the ANOVA I and Tukey’s post hoc test at significance levels of *p* < 0.05 (*), with respect to control.

**Figure 2:**
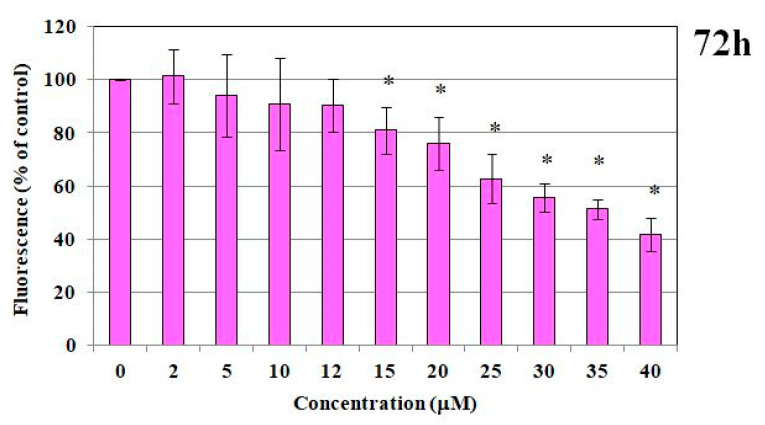
Assay of cell survival based on DNA content. Cell survival of the HMEC-1 cell line of PdTCEP was measured by Hoechst 33258 assay after 72 h exposure. Statistical evaluation of differences was made using the ANOVA I and Tukey’s post hoc test at significance levels of *p* < 0.05 (*), with respect to control.

The impact of PdTCEP on cell survival was evaluated. The PdTCEP demonstrated notable toxicity in the MTT test, particularly at concentrations exceeding 15 µM, when evaluated in immortalized human microvascular endothelial cells (HMEC-1) (Figure 1). The results obtained by the MTT assay were corroborated by the fluorimetric method (fluorescent probe Hoechst 33258) (Figure 2), which intercalates into DNA, thus enabling the viability and survival of cells to be analyzed based on DNA content. The addition of PdTCEP at a concentration of 15 µM resulted in a reduction in the survival of endothelial cells to approximately 80% in comparison to the control. In both tests, the greatest reduction in cell survival was observed for the highest concentration, namely 40 µM (40%). Additionally, statistically significant alterations in cell survival were discerned.

#### 2.1.2. ROS and RNS Production

**Figure 3:**
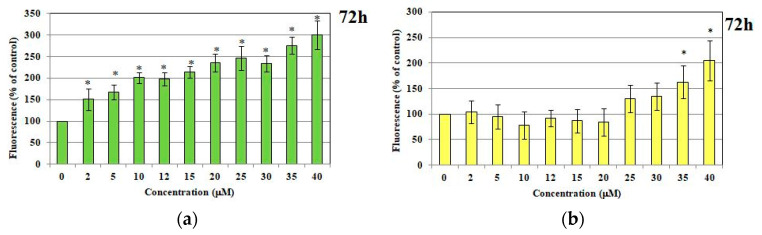
Intracellular reactive oxygen species (ROS) (**a**) and reactive nitrogen species (RNS) and (**b**) generation in HMEC-1 cells. Production of ROS and RNS of human dermal microvascular endothelial cell line after treatment of PdTCEP. Statistical evaluation of differences was made using the ANOVA I and Tukey’s post hoc test at significance levels of *p* < 0.05 (*), with respect to control.

The palladium(II) complex with tris(2-carboxyethyl)phosphine was observed to enhance the production of reactive oxygen species in human endothelial cells at 24, 48, and 72 h post-incubation. The level of ROS is the concentration-dependent on PdTCEP. If the concentration of PdTCEP is higher, the level of reactive oxygen species increases. It is visible at all concentrations, in particular after 72 h time incubation. The results demonstrated that PT-induced intracellular reactive oxygen species generation increased in a dose- and time-dependent manner (Figure 3a).

Following the incubation of cells with PdTCEP, the fluorescence intensity of products of DAF-FM-DA oxidation was increased, depending on PdTCEP concentration and time of exposure to endothelial cells. The effect was significant for the highest concentrations above 25 μM after 24, 48, and 72 h incubation (Figure 3b).

### 2.2. Red Blood Cells

#### 2.2.1. Hemolysis Assay

**Figure 4:**
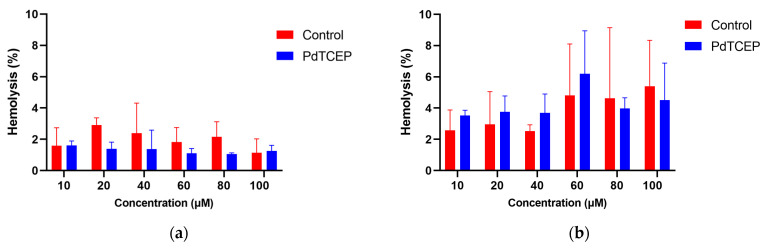
Percentage of hemolysis of red blood cells after 2 h (**a**) and 24 h (**b**) of incubation with PdTCEP at a temperature of 37 °C.

The second stage of the research was to determine the cytotoxic activity of PdTCEP against erythrocytes. Tests were carried out over a wide range of compound concentrations (0–100 μM) at different incubation times (Figure 4).

As the concentration of PdTCEP increased, a slight increase in the percentage of hemolysed cells was observed after a longer incubation time; this did not exceed the 10% level (Table S1). The study showed that the PdTCEP complex in the studied concentration range does not induce erythrocyte hemolysis, as according to the toxicity classification, compounds are nontoxic if the hemolysis rate is 0–9% [26].

#### 2.2.2. Shape of Erythrocytes

The likely location of the compound in the erythrocyte membrane can be determined by analyzing the shape changes in the erythrocytes. In the case of our results, we observed that with increasing concentration of the compound, more spherocytes (i.e., the uppermost forms of echinocytes) were formed, which was associated with a decrease in the number of discocytes at the same time (Table S2). According to the theory of Sheetz and Singer, the formation of echinocytes is due to the incorporation of compounds mainly into the outer lipid monolayer of the membrane, resulting in the outer layer increasing its surface area relative to the inner layer, which leads to outward deformation of the membrane and the formation of spicules. These results are in agreement with other authors and our previous work, in which it was found that if the concentration of echinocytogenic compounds is further increased, spherocytes begin to appear [26,27,28,29,30,31,32].

### 2.3. Influence of PdTCEP on the Selected Properties of the Membrane

#### 2.3.1. Steady-State Fluorescence

**Figure 5:**
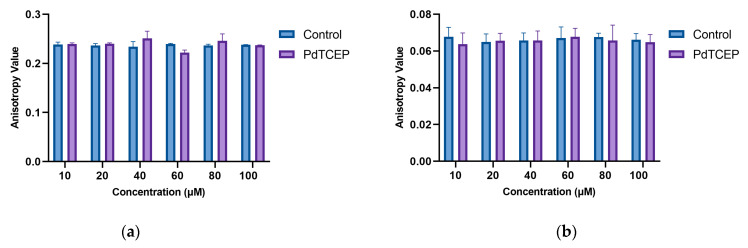
Values of fluorescence anisotropy of DPH probe for RBCMs (**a**) and ULVs (**b**) after 2 h incubation with the PdTCEP at 37 °C.

Measurements of membrane fluidity and the degree of ordering of the lipid bilayer were performed by fluorimetric method using the appropriately selected fluorescent probes DPH, Laurdan, and Prodan. The study was carried out for erythrocyte membranes (RBCMs) and liposome unilamellar formed from lecithin (ULVs). Measurements were made in the concentration range from 10 to 100 μM at a constant temperature of 37 °C (Figure 5). The results of anisotropy measurements for the DPH probe localized in the hydrophobic region of the lipid bilayer indicate that PdTCEP practically does not disrupt the structure of the model membrane in this region (Figure 5b). Slight changes in the anisotropy values are seen for compound concentrations above 40 µM for RBCMs (Figure 5a). Through changes in anisotropy, it is possible to detect whether the membrane fluidity of the biological membrane has been modified. The cell membrane is also significantly less fluid (A = 0.242 ± 0.004) compared to ULVs (A = 0.0678 ± 0.005). The slight differences in anisotropy changes for different PdTCEP-modified membrane models may indicate that there is a slightly different type of interaction between the compound and cell membrane components.

**Figure 6:**
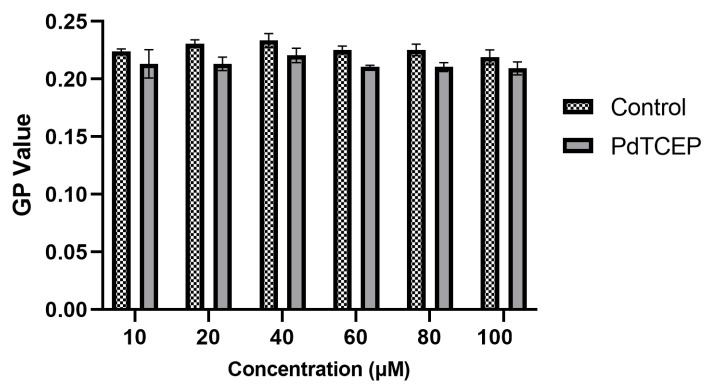
Values of fluorescence generalized polarization (GP) of Laurdan probe for RBCMs after 2 h incubation with PdTCEP at 37 °C.

The fluorescence of Prodan and Laurdan strongly depends on the polarity of the environment. Prodan, due to its slightly different molecular structure, differs from Laurdan in its site of distribution in the lipid membrane. This marker has a short propyl chain, which prevents it from anchoring in the hydrophobic part of the bilayer, and some of the probe molecules remain in solution. Laurdan has a long, 12-carbon aliphatic chain, which allows it to build into the non-polar interior of the lipid bilayer deeper than Prodan. Its fluorinating group is located at the level of lipid ester groups. When the membrane is in the gel phase, the emission maximum of Laurdan and Prodan is at 440 nm, and when the membrane is in the liquid crystalline phase, the emission maximum is at 490 nm. Generalized polarization (GP factor) was calculated by comparing the intensity of fluorescence at different emission wavelengths. The GP value indicates what is the accessibility of the probe’s fluorescent group’s surroundings to the water molecules in a given system (hydration). GP can therefore indicate how the polar heads of the lipid of the bilayer under study are arranged. Figure 6 compares GP values for RBCMs with DMSO and modified PdTCEP. As the concentration of the compound increases, the GP value decreases slightly, which may indicate a slight change in the ordering of the polar part of the bilayer.

**Figure 7:**
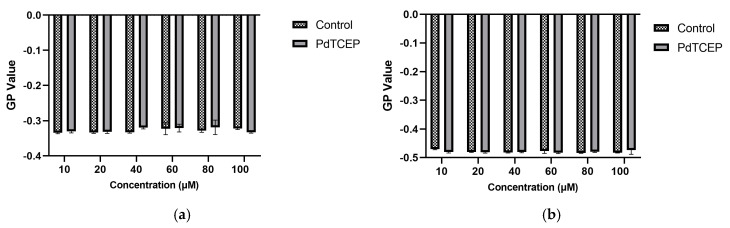
Values of generalized polarization (GP) of Laurdan (**a**) and Prodan (**b**) probes for liposomes (UVLs) modified with PdTCEP at 37 °C after 2 h.

A similar conclusion emerges from the results for the PdTCEP-modified liposome membrane for higher concentrations of the compound (Figure 7a). Interestingly, the GP coefficient for the Prodan probe did not change (Figure 7b). This would indicate that in the polar region, the membrane’s hydration does not change in the presence of PdTCEP.

#### 2.3.2. Attenuated Total Reflectance Infrared Spectroscopy Technique (ATR-FTIR)

**Figure 8:**
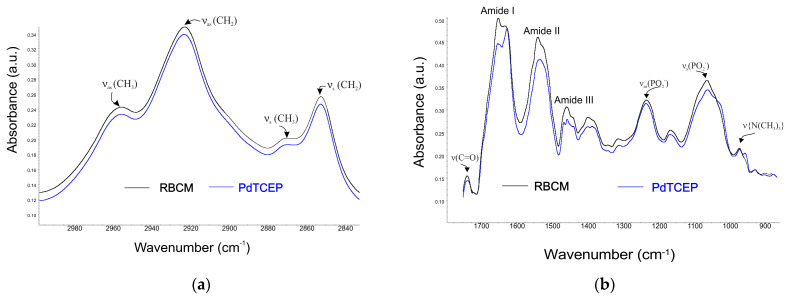
FTIR-ATR spectra of bands in the range of 3000–2840 cm^−1^ (**a**) and spectra of bands in the range of 1750–900 cm^−1^ (**b**) for the RBCMs measurements at 37 °C. Concentration of PdTCEP 80 µM.

In addition, FTIR was used to obtain more accurate data on the interaction of PdTCEP with erythrocyte membrane components. Structural changes in RBCMs were analyzed at the level of hydrophilic lipid groups, in the hydrophobic part, as well as in the lipid bilayer interface area and membrane proteins. Figure 8 shows a comparison of infrared spectra of RBCMs with DMSO and RBCMs doped with PdTCEP.

The most intense vibration in lipid systems is the CH_2_ stretching vibration and these give rise to bands in the 3000–2800 cm^−1^ region. The increase in the wave number of these bands testifies to an increase in fluidity of the hydrophobic part of the membrane. PdTCEP does not change the frequency of the signal from hydrocarbon chains (Figure 8). For this reason, we can assume that the tested compound does not significantly affect the hydrophobic regions of the lipid bilayer (Figure 8a). The interaction of PdTCEP and the head group of RBCM was monitored by analyzing the symmetric and asymmetric phosphate bands as well as choline stretching bands (Figure 8b). The frequency range of 1220–1260 cm^−1^ corresponds to the asymmetric stretching vibration of PO_2_^−^ phosphate groups. The ν_as_(PO_2_^−^) frequency band exhibits high sensitivity to changes in the environment polarity and to the possibility of interaction via hydrogen bonds. During the lipid bilayer hydration process, the number of phosphate groups interacting with water molecules increases and as a result, the maximum of the band moves towards lower wavenumbers. In the presence of palladium(II) complex, the frequency of oscillation is slightly shifted to higher values (for pure RBCMs the ν_as_(PO_2_^−^) = 1233.89), ν_as_(PO_2_^−^) = 1234.23, for PdTCEP. For symmetric vibration, the values of wavenumbers also change; ν_s_(PO_2_^−^) = 1062.63 for control and ν_s_(PO_2_^−^) = 1060.71 for PdTCEP. The changes in values of wavenumbers in the presence of compound indicate slight changes in the conformation of the polar lipid groups.

Additionally, in the presence of PdTCEP, the band of the phosphate group is much broader than for pure RBCMs, indicating that there is an interaction between the investigated compound and the lipid. The band observed in the polar part of the lipid spectra corresponds to the vibration in the choline fragment with the maximum at ν_as_(N−C) = 970.73 for control (RBCMs + DMSO). Analysis of the spectra showed that PdTCEP does not induce significant changes in this band. Significant changes were also not observed in the area of ester groups (1750–1700 cm^−1^). Visible changes in the presence of the tested compound, appear in the Amide I and Amide II band area, which may suggest that proteins present in the membrane have undergone some structural changes due to interaction with PdTCEP.

## 3. Discussion

Palladium(II) complex with tris(2-carboxyethyl)phosphine (PdTCEP) shows promising cytotoxic properties towards cancer cells. The present studies aimed to investigate the toxicity of PdTCEP towards normal human cells and its interaction with model cell membranes.

The research subjects were the human dermal microvascular endothelial cell line (HMEC-1) and red blood cells (RBCs), as well as the lipid–protein membranes of erythrocytes and model lipid membranes. It is of the utmost importance to conduct a comprehensive investigation into the interactions between novel compounds and blood components, given the potential for these compounds to be introduced into the body. It is established that compounds administered intravenously interact with the inner lining of blood vessels, thereby affecting the proper functioning of the vascular system (homeostasis). To maintain, for example, redox balance in cells, it is essential to investigate how oxidative stress caused by reactive oxygen species (ROS) and nitrogen species (RNS) impacts the cells. The levels of ROS and RNS in cells can be indicators of their health status and responses to various stressors. It is well known that an increase in ROS and RNS levels can result from the action of external factors, such as chemicals, toxins, and viral or bacterial infections, which can stimulate the body’s immune response. An increase in ROS levels indicates that the cells are undergoing oxidative stress, which can result in damage to proteins, lipids, and DNA. It is important to emphasize that an increase in ROS and RNS levels does not necessarily have to be negative; rather, its consequences depend on the context and how the cells respond to this increase. Thus, an elevation in their levels may be part of a normal cellular response to stress or changes in the environment. Reactive oxygen and nitrogen species can play a signaling role, influencing various cellular processes, including proliferation, differentiation, and apoptosis.

In a study using the HMEC-1 cell line, which represents healthy endothelial cells, the toxicity of the compound PdTCEP was evaluated. The results showed that an increase in PdTCEP concentration was associated with a higher rate of cell death. At a concentration of 15 µM, cell viability decreased to approximately 80%, while at 40 µM, the most significant decline was observed, dropping to 40%. The studies demonstrated a beneficial effect of PdTCEP on ROS levels, as an increase in ROS levels was only detected after 72 h of incubation. This suggests that the toxic effect of the compound may be related to its long-term action on cells rather than a direct impact on their viability in the short term.

The assessment of toxicity towards erythrocytes is of fundamental importance in the context of the potential use of compounds as pharmaceuticals. Erythrocytes are responsible for delivering oxygen to cells as well as removing carbon dioxide from the lungs. They have a discocyte shape, which can change under the influence of various physical and chemical factors. The reversible transformation of discocytes into stomatocytes can be triggered by amphipathic substances that accumulate in the inner monolayer of the lipid bilayer in the cell, acidic pH, and high hydrostatic pressure. On the other hand, the transformation of discocytes into echinocytes is possible if amphipathic compounds accumulate in the outer monolayer of the cell’s lipid bilayer; such shape changes can also occur due to lower ATP levels, excessive cholesterol, and alkaline pH. The hemolytic activity of PdTCEP was tested over a wide range of compound concentrations from 10 to 100 μM. No hemolysis was observed in samples incubated for one hour, and similar results were obtained after 2 and 24 h of incubation at 37 °C. The percentage of hemolysis calculated for PdTCEP remained mostly at a level comparable to the control, even for high concentrations of the compound. Our studies indicate that PdTCEP does not induce hemolysis of red blood cells, suggesting that the compound does not cause damage to the cell membrane. Furthermore, based on optical microscopy images, it was found that PdTCEP causes a change in the shape of erythrocytes from discoid to echinocytic. According to the theory of coupled monolayers, this indicates that the compound likely affects the outer layer of the erythrocyte membrane. This leads to an increase in the outer surface area of the red blood cell membrane in relation to the inner surface. The monolayer undergoes deformation, resulting in the characteristic protrusions that were observed under the microscope. If the compound were acting on the inner monolayer, the erythrocytes would take on a stomatocytic shape with a cup-like appearance, which has also been shown by numerous researchers for other compounds [27,28,29,30,31,32].

Additionally, studies were conducted on the effect of PdTCEP on the properties of lipid-protein membranes isolated from erythrocytes (RBCMs) and, for comparison, on model lipid membranes (unilamellar vesicles—ULVs). The cell membrane is an extremely important component of erythrocytes due to its many functions. The types of lipids present in the cell membrane of erythrocytes are primarily cholesterol, phospholipids, and glycolipids. Due to the diversity in both the structure and chemical composition of biological membranes, analyzing the structural and physicochemical changes in such membranes is complex. Therefore, it is reasonable to create simplified models of the cell membrane, among which unilamellar vesicles are the most commonly used [33,34]. Studies on the fluidity and degree of ordering of RBCMs and ULVs were conducted using fluorescent probes such as DPH, Laurdan, and Prodan. These markers were chosen due to their different localization sites in the biological membrane. The DPH probe effectively illustrates disturbances in the hydrophobic area of the bilayer and emits fluorescence from the region where the alkyl chains of membrane lipids are located. For this reason, DPH was used to assess membrane fluidity based on the analysis of changes in anisotropy in this region of the bilayer. The increase in fluorescence anisotropy is related to the restriction of the rotational motion of the marker, and thus to the degree of packing of hydrocarbon chains and, consequently, to the increase in the rigidity of the biological membrane. A decrease in the value of parameter A indicates an increase in membrane fluidity, which can be explained by structural changes in the hydrophobic region of the bilayer, likely due to the interaction of the studied substances with lipids [34]. The anisotropy value slightly decreased in the case of RBCMs modified with PdTCEP, indicating a slight increase in the fluidity of erythrocyte membranes. One of the functions of cell membranes is the regulation and control of the transport of substances into and out of the cell. When these transport mechanisms are unstable, the cell is susceptible to damage. Furthermore, a decrease in the fluidity of the cell membrane in the erythrocytes can disrupt the proportion of the composition of both bilayers and impede the shape change in erythrocytes, which is extremely important in red blood cells due to microcirculation in the blood vessels. For liposomes, the anisotropy value practically did not change. The differences in anisotropy values are most likely due to the different composition of the membranes. The membrane of erythrocytes is much stiffer since, unlike the lipid model membrane of liposomes, it also contains proteins, cholesterol, and a diverse lipid composition. Liposomes (ULVs) were formed from egg phosphatidylcholine. Changes in the order of model membranes were examined using the Laurdan and Prodan probes. Both probes are sensitive to changes in the polarity of the surrounding environment. Laurdan is situated in the hydrophilic-hydrophobic area of the bilayer, with the lauric acid tail anchored in the acyl chain region of the phospholipids and the naphthalene ring (the fluorescent element) located at the glycerol level in the lipid bilayer [35]. Changes occurring in the hydrophilic part of the membrane induced by the addition of PdTCEP were determined based on the calculated general polarization (GP) coefficient of the Laurdan probe. A decrease in the GP value under the influence of compounds modifying the bilayer may indicate an increase in “disorder” in the polar area of the lipids and their greater dynamics, while an increase in GP is associated with increased ordering and/or a smaller content of water molecules [36]. A slight decrease in GP values was observed for both RBCMs and liposomes (to a lesser extent) at higher concentrations of PdTCEP (above 40 µM). PdTCEP thus slightly contributes to changes in the ordering of the hydrophilic part of the lipid bilayer, likely interacting only with the polar part of the cell membrane. Similar results were obtained for the platinum complex with tris(2-carboxyethyl)phosphine. A more detailed analysis of interactions with the phospholipid bilayer showed that the platinum(II) complex interacts with the polar groups of the lipid not only through electrostatic forces but also forms hydrogen bonds with the polar heads of the phospholipids [19]. Additionally, it increases the ordering of the hydrophobic part of the bilayer and slightly decreases the fluidity of the membrane, which may be crucial for the proper function of membrane-associated proteins. This would be consistent with the results obtained for PdTCEP, as infrared spectroscopy analysis for RBCMs indicates that structural changes in membrane proteins (regions Amide I and Amide II) may occur under the influence of PdTCEP [26].

In summary, PdTCEP exerts a slight influence on the structure of erythrocyte membranes and lipid membranes. These changes are slightly different in erythrocyte membranes and lipid membranes, suggesting that the effect of the palladium complex was not limited to the lipids present in the studied systems. The presented studies also demonstrated that PdTCEP affects the human microvascular endothelial cell line (HMEC-1), but does not exhibit toxicity towards erythrocytes, and does not cause cell membrane destruction. Auto-metathesis, in which ligand exchange occurs [20], may suggest that, under biological conditions, similar reactions may occur involving natural ligands, such as amino acid carboxyl groups, which may act as ligands in metal complexes. Our initial hypothesis is that palladium may exist in the cellular environment in the form of complexes with biological ligands, which may have important implications for understanding its role in potential therapeutic applications.

## 4. Materials and Methods

### 4.1. Materials

The palladium(II) complex with tris(2-carboxyethyl)phosphine: trans-[PdCl_2_ {P(C_2_H_4_COOH)_3_}_2_] (PdTCEP) was prepared by the literature procedures described earlier [20]. Fluorescent probes (DPH, Prodan, and Laurdan), and dimethylformamide (DMF) for dissolving probes, were bought in Merck (Darmstadt, Germany). Ethanol and DMSO was bought from Avantor Performance Materials, Gliwice, Poland.

The research was conducted on human red blood cells (RBC) obtained from a blood center from examined and healthy donors and isolated red blood cell membranes (RBCMs). RBCMs were obtained from blood using the method described by Dodge et al. [37] with minor modifications [38]. According to Polish law, the use of erythrocytes from blood centers in experiments does not require approval from an ethics committee. Fresh blood was suspended in a physiological saline solution with added heparin each time. The lipids from the red blood cells were extracted from the erythrocyte membranes according to the method described by Maddy et al. [38]. Liposomes (ULVs) were formed using egg lecithin purchased from Sigma Aldrich (St. Louis, MO, USA).

### 4.2. Cell Line

The human dermal microvascular endothelial cell line (HMEC-1) was obtained from the American Type Culture Collection (ATCC, CRL-3243, Dziekanów Leśny, Poland). The cells were maintained in MCDB 131 medium supplemented with 10% fetal bovine serum (FBS), 10 mM L-glutamine, 1 μg/mL hydrocortisone, 1% penicillin/streptomycin, and 10 ng/mL epidermal growth factor (EGF). Cultures were grown under standard conditions (5% CO_2_, 37 °C) in plastic flasks. The seeding density was selected to ensure logarithmic cell growth up to the time of analysis.

### 4.3. Cytotoxicity Test of Normal Cells

The impact of PdTECP on HMEC-1 cell proliferation was assessed using the MTT assay, which measures the reduction in the tetrazolium dye 3-(4,5-dimethylthiazol-2-yl)-2,5-diphenyltetrazolium bromide (MTT) into insoluble formazan by viable cells. HMEC-1 cells were seeded in 96-well plates at a density of 5000 cells per well and cultured for 12–24 h. After this initial period, silica nanoparticles were introduced at concentrations ranging from 0 to 40 µM, and the incubation continued for 24, 48, or 72 h. Following the incubation, the cell monolayers were washed twice with Hanks’ balanced salt solution (HBSS) and fresh medium containing 20 µL of MTT solution (5 mg/mL) was added. After a 2-h incubation period, the medium was discarded, and the resulting formazan crystals were dissolved in DMSO. The absorbance of the dissolved formazan was measured at 570 nm, with the values serving as an indicator of the number of viable cells in each sample.

### 4.4. Assay of Cell Survival Based on DNA Content

For the subsequent cytotoxicity assessment focusing on DNA content, the fluorescent probe Hoechst 33258 was employed. HMEC-1 cells were seeded in a 96-well black plate at a density of 5000 cells per well and incubated at 37 °C under 5% CO_2_. The cells were treated with PdTCEP at concentrations ranging from 0 to 40 µM and incubated for 24, 48, and 72 h. After the incubation period, the medium was carefully aspirated, and the cells were washed twice with HBSS. The plate was then frozen at −70 °C. In the next step, the cells were thawed at room temperature, followed by the addition of 100 µL of deionized water to each well. The plate was frozen again at −70 °C. After a second thawing, 100 µL per well of HVAB solution (0.5% Hoechst 33258 in TNE buffer containing 2 M NaCl, 1 mM EDTA, and 10 mM Tris–HCl, pH 7.4) was added. The plate was gently shaken and incubated in the dark at room temperature for 15 min. Fluorescence intensity was then measured at excitation/emission wavelengths of 355/460 nm.

### 4.5. Determination of ROS and RNS Production

The production of reactive oxygen and nitrogen species (ROS/RNS) was evaluated in HMEC-1 cells seeded in 96-well black plates at a density of 5000 cells per well and cultured for 24 h. PdTCEP was then added at various concentrations, and the cells were incubated for 24, 48, and 72 h under standard conditions. Following incubation, the cell monolayers were washed with HBSS, and a fluorogenic probe was introduced: either 5 µM 2′,7′-dichlorodihydrofluorescein diacetate (H2DCF-DA) or 5 µM 3-amino-4-aminomethyl 2′,7′-dichlorofluorescein diacetate (DAF-FM-DA) in HBSS. After a 2-h incubation in the dark at 37 °C under 5% CO_2_, fluorescence was measured at λ_ex_ = 485 nm and λ_em_ = 538 nm for H2DCF-DA, and at λ_ex_ = 485 nm and λ_em_ = 510 nm for DAF-FM-DA.

Subsequently, DNA content was assessed. After measuring ROS and RNS, the cells were frozen at −70 °C. Upon thawing, 100 µL of distilled water was added to each well, and the cells were refrozen. Following a second thaw, 50 µL of 15 µg/mL ribonuclease A was added to each well and incubated for 30 min in the dark at 37 °C. Next, 50 µL of 10 µM propidium iodide (PI) in deionized water was added, followed by a 15-min incubation under the same conditions. The fluorescence intensity of PI was then measured at λ_ex_ = 355 nm and λ_em_ = 620 nm.

The rate of ROS/RNS production was calculated using the following formula:

$$Rate\;of\;ROS/RNS=\frac{intensity\;of\;H2DCF\text{-}DA/DAF\text{-}FM\text{-}DA\;fluorescence}{intensity\;of\;PI\;fluorescence}$$

The fluorescence value of the control samples was set as 100%.

### 4.6. Hemolysis of Erythrocytes

The hemolytic test was performed following the method described by Pruchnik et al., with slight modifications [26]. Whole blood was centrifuged at 2500 rpm for 3 min at 4 °C to separate plasma and leukocytes. The erythrocytes (RBCs) were then washed three times with cold phosphate-buffered saline (PBS) of isotonic strength (pH 7.4, 310 mOsm). Test samples (1 mL) were prepared by mixing an appropriate volume of buffer solution, the PdTCEP compound, and erythrocytes to achieve a final hematocrit of 1.2%. Hemolytic activity of the complex was evaluated at concentrations ranging from 0 to 100, following incubation at 37 °C for 2 and 24 h. After incubation, 2 mL of PBS (pH 7.4) was added, and the samples were centrifuged at 2500 rpm for 15 min at room temperature. The hemoglobin content in the supernatant was measured using a UV-Vis spectrophotometer (Specord 40, Analytik Jena, Jena, Germany) at 540 nm. The hemoglobin concentration in the supernatant, expressed as a percentage of the concentration in completely hemolyzed cells, was used to assess the degree of hemolysis. Complete hemolysis (100%) was achieved by adding distilled water to the samples.

### 4.7. Shape of Erythrocytes

The shape of red blood cells modified with PdTCEP was investigated using the optical microscopes (Nikon Eclipse E200, Nikon, Tokyo, Japan). The method was described earlier in Pruchnik et al. [26]. This method allows us to determine the localization of compounds in the erythrocyte membrane based on changes in erythrocyte shapes. For investigation with the optical microscope, the red cells were prepared as described in Section 4.6. The cells were then suspended in a 0.9% NaCl solution so that the final hematocrit of the cells in the sample was 1.2%. The compound was then added at concentrations of 20 and 40 µM. After that, the samples were incubated for 1 h at 37 °C. The shapes of erythrocytes have been described according to the Bessis and Brecher scale—in which three basic shapes, (0) discocytes, (−) stomatocytes, and (+) echinocytes, are assigned appropriate morphological indices.

### 4.8. Influence of PdTCEP on the Selected Properties of the Membrane

#### 4.8.1. Steady-State Fluorescence Spectroscopy

In this study, the effect of PdTCEP on the properties of RBCMs and ULV liposomes was investigated. RBCMs content in each sample was standardized using protein concentration, measured by the Bradford method [39]. Lipid concentration was fixed at 0.1 mg/mL. Three fluorescent probes, Laurdan, Prodan, and DPH, were employed at 1 µM concentrations. For preparation, RBCMs or liposomes and fluorescence probes were suspended in an isotonic phosphate solution with pH 7.4. This suspension was incubated for 30 min in the dark at room temperature. Subsequently, the mixtures were portioned into cuvettes, and compounds were added in concentrations ranging from 5 to 100 µM. These samples underwent incubation for one hour at 37 °C, followed by fluorimetric analysis after an additional two-hour period. Measurements were carried out using a Cary Eclipse fluorimeter (Varian, Palo Alto, CA, USA) at 37 °C. For DPH, excitation and emission wavelengths were 360 nm and 425 nm, respectively. Based on the changes in DPH intensity under polarized light, the anisotropy value (A) was determined according to the formula used in our previous publications [26,34]. For Laurdan and Prodan, excitation was set at 360 nm, with emissions recorded at 440 nm and 490 nm. Generalized polarization (*GP*) values for Laurdan and Prodan were calculated using the following formula:

$$GP=\frac{I_{b}-I_{r}}{I_{b}+I_{r}}$$

where *I_b_* is fluorescence intensity at λ = 440 nm, and *I_r_* is fluorescence intensity at λ = 490 nm.

#### 4.8.2. Attenuated Total Reflectance Fourier Transform Infrared Spectroscopy (ATR-FTIR)

The Fourier Transform Infrared (FTIR) spectroscopy method was applied to study the interactions between compounds and specific functional groups of lipids, such as choline, carbonyl, phosphate, and hydrocarbon chains. Additionally, it examined protein groups, specifically Amide I and Amide II, within RBCMs. Detailed protocols for preparing RBCMs are available in earlier work [26]. In brief, after removing water from the samples, spectra of both control (RBCMs) and test samples (at a concentration of 80 µM) were recorded. Measurements were conducted at 37 °C using a Thermo Nicolet 6700 MCT spectrometer (Thermo Fisher Scientific, Waltham, MA, USA). Each spectrum was derived from 128 scans at a resolution of 2 cm_−1_, covering the spectral range of 700–4000 cm_−1_. Preliminary data analysis was carried out using the EZ OMNIC v 8.0 software.

## 5. Conclusions

The study of interactions between new compounds and endothelial cells and red blood cells is crucial for understanding their potential effects in the context of therapy or the introduction of new drugs into the body. Our studies have shown selective effects of PdCTEP on erythrocytes and vascular endothelial cells of the skin, confirming the validity of further tests of the palladium–phosphine complex as a potential anticancer agent in relation to cancer cell lines. Based on the results, we can conclude that in a wide range of tested concentrations, PdTCEP does not cause destruction of the erythrocyte membrane and has a slight effect on the fluidity of the lipid and lipid–protein model membrane. However, it significantly affects the survival of HMEC-1. Increasing the concentration of the compound initiates oxidative stress and increases the level of reactive nitrogen species, which can affect various cellular processes, including cell proliferation, differentiation, or apoptosis. Therefore, PdTCEP is a promising compound for further biological and pharmacological testing.

## Data Availability

No new data were created or analyzed in this study. Data sharing is not applicable to this article.

## Supplementary Materials

The following supporting information can be downloaded at: https://www.mdpi.com/article/10.3390/molecules30030476/s1, Table S1. Comparison of the percentage of hemolysis of erythrocytes modified with palladium(II) complex for two incubation times (1 h and 24 h). Table S2. Mean percentage of erythrocyte shapes formed in the presence of PdTCEP compound applied at 20 μM and 40 μM.
