# Supplementary material for: Impact of a Palladium(II)-tris(2-carboxyethyl)phosphine Complex on Normal Cells: Toxicity and Membrane Interaction
Source: Molecules. 2025 Jan 22;30(3):476. doi: 10.3390/molecules30030476 (PMC11821180; doi:10.3390/molecules30030476)
Supplement: Supplementary file 1 [file molecules-30-00476-s001.zip › molecules-3384150-supplementary.pdf]

Supplementary material:

Table S1. Comparison of the percentage of haemolysis of erythrocytes modified with palladium(II) complex for two incubation times (1h and 24-h).

Concentration	Control	PdTCEP	Control	PdTCEP
	1h		24 h	
**10**	1.58± 0.98	1.60± 0.29	2.58± 0.90	3.53± 0.33
**20**	2.91± 0.46	1.38± 0.44	2.96± 0.46	3.76± 1.02
**40**	2.39± 0.91	1.38± 0.93	2.52± 0.41	3.24± 0.91
**60**	1.82± 0.93	1.11± 0.30	4.82± 1.93	6.20± 1.81
**80**	2.16± 0.90	2.05± 0.08	4.63± 1.90	3.94± 0.69
**100**	1.14±0.85	1.25±0.37	5.40±1.89	4.51±1.90

Table S2. Mean percentage of erythrocyte shapes formed in the presence of PdTCEP compound applied at 20 μM and 40 μM.

	Control	20 μM	40 μM
**Shape of erythrocytes**	Average percent share of individual forms of erythrocytes ± SD		
**Spherostomatocytes (-4)**	6.78± 0.82	10.24± 0.54	4.64± 0.78
**Stomatocytes II (-3)**	6.49± 0.98	7.99± 1.05	7.67± 0.85
**Stomatocytes I (-2)**	3.83± 0.54	12.30± 0.48	12.33± 0.57
**Discostomatocytes (-1)**	3.54± 0.37	10.24± 0.35	18.36± 0.43
**Discocytes (0)**	22.14± 0.36	16.53± 0.99	4.11± 0.145
**Discoechinocytes (1)**	14.75± 0.37	9.92± 0.48	4.11± 0.27
**Echinocytes (2)**	16.22±0.85	9.09±0.54	11.51±0.65
**Spheroechinocytes (3)**	15.63±0.67	12.67±0.68	9.32±0.35
**Spherocytes (4)**	10.62±0.43	11.02±0.88	27.95±1.15
